# Supplementary material for: Towards Cohesive National Surveys in Pakistan: A Comparative Study of DHS and PSLM
Source: PLoS One. 2025 Mar 25;20(3):e0320044. doi: 10.1371/journal.pone.0320044 (PMC11936229; doi:10.1371/journal.pone.0320044)
Supplement: S1 File — (DOCX) [file pone.0320044.s001.docx]

**Human Participants Research Checklist**

***Complete the following if your study involved human participants or human participants’ data. These questions should be addressed for prospective and retrospective studies.***

1. Did you obtain ethics approval for this study?
   - If yes, please upload (file type “Other”) the original approval document you received from your ethics committee. If the original document is in another language, please also provide an English translation.

___ Uploaded _x_ N/A

- - If you did not obtain ethical approval, please explain why this was not required below.

We used secondary data from national survey Pakistan Social and Living Measurement (PSLM) which is collected by government of Pakistan and it is publicly available data on Pakistan Bureau of Statistics (PBS) website and Pakistan Demographic and Health Survey which was conducted by National Institute of Population Studies (NIPS), Pakistan. As we are using the secondary data, we did not require any ethical approval.

1. If you prospectively recruited human participants for the study – for example, you conducted a clinical trial, distributed questionnaires, or obtained tissues, data or samples for the purposes of this study, please report in the Methods:
   1. the day, month and year of the **start and end** of the recruitment period for this study.
   2. whether participants provided informed consent, and if so, what type was obtained (for instance, written or verbal, and if verbal, how it was documented and witnessed). If your study included minors, state whether you obtained consent from parents or guardians. If the need for consent was waived by the ethics committee, please include this information.

___ Completed _x_ N/A

1. If you are reporting a retrospective study of medical records or archived samples, please report in the Methods section:
2. the day, month and year when the data were accessed for research purposes
3. whether authors had access to information that could identify individual participants during or after data collection

___ Completed _x_ N/A
