# Supplementary material for: Towards Cohesive National Surveys in Pakistan: A Comparative Study of DHS and PSLM
Source: PLoS One. 2025 Mar 25;20(3):e0320044. doi: 10.1371/journal.pone.0320044 (PMC11936229; doi:10.1371/journal.pone.0320044)
Supplement: S1 Data — (DOCX) [file pone.0320044.s002.docx]

# **S1 Data:** The table below shows the comparison of questions asked in both PDHS PSLM surveys

**Table 4: Family Planning Questions**

| Family Planning Indicators | PDHS | PSLM |
| --- | --- | --- |
| Have you ever heard of any method? | 🗸 | 🗸 |
| Did you hear about family planning method before your marriage? | 🗸 | ✗ |
| Are you currently using any family planning method? | 🗸 | 🗸 |
| Which method are you currently using? | 🗸 | 🗸 |
| What is the brand name of the pills you are using? | 🗸 | ✗ |
| What is the brand name of the condom you are using? | 🗸 | ✗ |
| In what facility did the sterilization take place? | 🗸 | ✗ |
| In what month and year was the sterilization performed? | 🗸 | ✗ |
| Since what month and year have you been using current method without stopping? | 🗸 | ✗ |
| How many times you or your husband may have used a method to avoid getting pregnant during last few years? | 🗸 | ✗ |
| When was the last time you used a method? Which method was that? When did you start using that method? How long after the birth of last child did you use the method? | 🗸 | ✗ |
| Why did you stop using that method? | 🗸 | 🗸 |
| Did you become pregnant while using (method), or did you stop to get pregnant, or did you stop for some other reason? | 🗸 | ✗ |
| Have you ever used anything or tried in any way to delay or avoid getting pregnant? | 🗸 | 🗸 |
| Where did you get it [current method] at that time? | 🗸 | 🗸 |
| At that time, were you told about side effects or problems you might have with the method? | 🗸 | ✗ |
| When you got sterilized, were you told about side effects or problems you might have with the method? | 🗸 | ✗ |
| Were you ever told by a health or family planning worker about side effects or problems you might have with the method | 🗸 | ✗ |
| Were you told what to do if you experienced side effects or problems? | 🗸 | ✗ |
| Were you advised by a health or family planning worker about the following:  Help you in selecting a method?  Were you ever told by a health or family planning worker about side effects or problems you might have with the method?  Explained how to use the selected method? | 🗸 | ✗ |
| Where did you obtain (current method) the last time? | 🗸 | ✗ |
| Do you know of a place where you can obtain a method of family planning? | 🗸 | ✗ |
| Did the LHW talk to you about family planning? | 🗸 | ✗ |
| In the last 12 months, have you visited a health facility for care for yourself or your children? | 🗸 | ✗ |
| Did any staff member at the health facility speak to you about family planning methods? | 🗸 | ✗ |
| Any birth or pregnancy termination after month and year of start of use of contraception | 🗸 | ✗ |
| Are you satisfied with this method of family planning? | ✗ | 🗸 |
| Why you are not satisfied with this method of family planning? | ✗ | 🗸 |
| After the child you are now expecting, would you like to have another child, or would you prefer not to have any more children? | 🗸 | 🗸 |
| Would you like to have (a/another) child, or would you prefer not to have any (more) children? | 🗸 | 🗸 |
| How long would you like to wait before the birth of (a/another) child? | 🗸 | 🗸 |

**Table 5: Child Immunization Questions**

| Child Immunization Indicators | PDHS | PSLM |
| --- | --- | --- |
| Were you vaccinated for BCG? | 🗸 | 🗸 |
| Were you vaccinated for Penta 2 | 🗸 | 🗸 |
| Were you vaccinated for Penta 3 | 🗸 | 🗸 |
| Were you vaccinated for Pneumococcal 1 | 🗸 | 🗸 |
| Were you vaccinated for Pneumococcal 2 | 🗸 | 🗸 |
| Were you vaccinated for Pneumococcal 3 | 🗸 | 🗸 |
| Were you vaccinated for Polio 1 | 🗸 | 🗸 |
| Were you vaccinated for Polio 2 | 🗸 | 🗸 |
| Were you vaccinated for Polio 3 | 🗸 | 🗸 |
| Were you vaccinated for Measles 1 | 🗸 | 🗸 |
| Were you vaccinated for Measles 2 | 🗸 | 🗸 |
| Were you vaccinated for ROTAVIRUS 1 | 🗸 | ✗ |
| Were you vaccinated for ROTAVIRUS 2 | 🗸 | ✗ |
| Were you vaccinated for ROTAVIRUS 3 | 🗸 | ✗ |
| What date the most recent immunization was given? | 🗸 | 🗸 |
| Where the most recent immunization was given? | ✗ | 🗸 |
| How many days after birth, did the child get first injection of BCG? | 🗸 | 🗸 |
| How far did you travel (round trip) to get immunization? | ✗ | 🗸 |
| How much did you pay for it? | ✗ | 🗸 |
| Why was the child not immunized? | ✗ | 🗸 |
