# Supplementary material for: Towards Cohesive National Surveys in Pakistan: A Comparative Study of DHS and PSLM
Source: PLoS One. 2025 Mar 25;20(3):e0320044. doi: 10.1371/journal.pone.0320044 (PMC11936229; doi:10.1371/journal.pone.0320044)
Supplement: Attachment — Submitted filename: Reviewers Comments.docx [file pone.0320044.s003.docx]

| Reviewers’ Comments | Response |
| --- | --- |
| Reviewer #1: Having read through the submission, i see the paper as a vital tool for future programming and an addition to the body of knowledge. This paper once accepted will help in decision making in Pakistan and globally. | Thank you for your comments, really appreciate that you acknowledge the efforts from the authors |
| Reviewer #2: As a reviewer, I find this article to be logically ordered, well-structured and presented succintly and clearly. The similarities and complexities of the various surveys are well-presented. Save from a few spelling omissions e.g. “in depth (sic)”, the paper is written in a scholarly, yet readable and accessible format.  Some proposed revisions are regarding uniformity and consistency in the number of significant figures e.g. in Table 4 which has both 3 and 4 significant figures and both 1 or 2 decimal places. Table 2 is particularly hard to read in a “wide” format, and a long format is suggested, including disaggregation by provinces of the number of respondents. It would also be helpful to include the number of children for which the MWRA respondents reported, especially given that immunization is a major component of the study. Additional proposed additions to the details on the rudiments of the two surveys include mentioning or clarifying the specific reference or recall periods for the indicators.  The authors could also make a good case advocating for efforts to harmonize survey methodologies, and key indicators across these different survey types. While the authors report differences in respondent selection for certain question types and outbreaks before one of the surveys that resulted in differences, there could be more clarification on immunization indicators in children diverging so much between the two questionnaires and the implications of such, given that both surveys are provincially representative and country-wide potentially even-ing out the impact of the regional immunization activities.  Have concordance surveys taken place between DHS and national surveys in other countries? MICS and DHS? A global perspective would be helpful to add in the discussion.  Overall, a solid paper that discusses a critical need. | Table 4 has been corrected, now all figures are 2 decimal places.  Table 2 has been corrected with additional information on the immunization section, sample sizes of children 12 – 23 months age and 24 – 35 months age.  Comparison between MICS and DHS globally reference has been added under immunization section discussion. Reference point of postnatal comparison has been used to link it with recall bias. Thank you for highlighting this additional point for our assistance. |
